# Genome-wide analysis of epigenetic dynamics across human developmental stages and tissues

**DOI:** 10.1186/s12864-019-5472-0

**Published:** 2019-04-04

**Authors:** Xia Zhang, Yanglan Gan, Guobing Zou, Jihong Guan, Shuigeng Zhou

**Affiliations:** 1 School of Computer Engineering and Science, Shanghai University, Shanghai, China; 2 School of Computer Science and Technology, Donghua University, Shanghai, China; 3 Department of Computer Science and Technology,Tongji University, Shanghai, China; 4 Shanghai Key Lab of Intelligent Information Processing, and School of Computer Science, Fudan University, Shanghai, China

**Keywords:** Epigenetic modification, Differential analysis, Hamming distance

## Abstract

**Background:**

Epigenome is highly dynamic during the early stages of embryonic development. Epigenetic modifications provide the necessary regulation for lineage specification and enable the maintenance of cellular identity. Given the rapid accumulation of genome-wide epigenomic modification maps across cellular differentiation process, there is an urgent need to characterize epigenetic dynamics and reveal their impacts on differential gene regulation.

**Methods:**

We proposed DiffEM, a computational method for differential analysis of epigenetic modifications and identified highly dynamic modification sites along cellular differentiation process. We applied this approach to investigating 6 epigenetic marks of 20 kinds of human early developmental stages and tissues, including hESCs, 4 hESC-derived lineages and 15 human primary tissues.

**Results:**

We identified highly dynamic modification sites where different cell types exhibit distinctive modification patterns, and found that these highly dynamic sites enriched in the genes related to cellular development and differentiation. Further, to evaluate the effectiveness of our method, we correlated the dynamics scores of epigenetic modifications with the variance of gene expression, and compared the results of our method with those of the existing algorithms. The comparison results demonstrate the power of our method in evaluating the epigenetic dynamics and identifying highly dynamic regions along cell differentiation process.

**Electronic supplementary material:**

The online version of this article (10.1186/s12864-019-5472-0) contains supplementary material, which is available to authorized users.

## Background

Lineage specification and maintenance of cellular identity are complex biological processes [1]. It is now widely accepted that cell phenotypes are significantly regulated by epigenetic states and that chromatin changes during differentiation contribute to the determination of cell fate [2]. Recent evidence further shows that coordinated epigenetic changes influence the maintenance of such cellular memory [3, 4]. DNA methylation and certain epigenetic modifications are essential for chromatin structures and gene expression in proper execution of developmental programs [5, 6]. Therefore, a fundamental question in the field is to exactly answer where and how the epigenetic changes regulate phenotypic changes.

To fully understand the dynamics and regulatory roles of epigenetic modifications, advanced sequencing technologies have generated genome-wide epigenetic maps of diverse developmental stages, lineages and tissues [7, 8]. In previous studies, researchers have differentiated human embryonic stem cells (hESCs) into mesendoderm, neural progenitor cells, trophoblast-like cells, and mesenchymal stem cells and systematically sequenced the transcriptome and epigenetic modifications of these lineages [9, 10]. The first three hESC derivatives reflects critical developmental linages in the embryo [11]. Mesenchymal stem cells have the ability of further multi-lineage differentiation to bone, cartilage, adipose, muscle, and connective tissues [12]. Mouse embryonic stem cells were also differentiated into a variety of precursor cell types [13]. The expanding body of epigenomic data permits researchers to study the dynamics of epigenetic marks. This is a key step to reveal regulatory roles of epigenetic modifications, and to understand how global features of epigenetic modifications impact cellular phenotypes across different developmental stages, lineages and tissues.

Most previous works focused on comparing the epigenetic modification profiles between two biological conditions, and further identifying regions that show differential patterns, such as ChIPDiff [14], diffReps [15], dPCA [16], HistoneHMM [17], csaw [18] and HMCan-diff [19]. While some other methods such as dMCA [20] and Yang’s method [21], were designed to detect cell-type-specific differential regions. Moreover, there are also some methods that were designed for identifying differential methylated region, such as QDMR [22] and MethylAction [23], whereas QDMR can also be applied to histone modification data analysis. Although several algorithms have been developed to analyze the epigenetic difference between two different conditions, little work devoting to differential analysis of epigenetic modifications among multiple cell types and across different developmental stages.

Here, we presented DiffEM, a computational method to quantify the dynamics of epigenetic marks and identified highly dynamic modification sites (HDMSs) across different human embryonic developmental stages. We applied this method to a public datasets with 6 intensely studied epigenetic marks of 20 different developmental stages and tissues. We identified HDMSs where different cell types exhibit distinctive epigenetic modification patterns, and found that these highly dynamic sites are enriched in genes related to cellular development and differentiation. We further correlated the dynamics scores of these epigenetic marks with those of gene expression levels. The results indicate that the changes of gene expression are closely related to the modification patterns of H3K4me1 and H3K27me3 in promoter regions during cell differentiation process. We compared DiffEM with the existing algorithms for identifying HDMSs. The comparison results show that DiffEM perform better in evaluating the epigenetic dynamics and identifying highly dynamic modification sites. This method is promising for broad applications in evaluating epigenetic dynamics in other complex biological processes.

## Materials and methods

### Datasets

To analyze the dynamic epigenetic changes during cellular differentiation and lineage specification, we obtained a large panel of epigenetic maps of human embryonic stem cells (hESCs) and the key derivatives, including trophoblast-like cells (TBL), mesendoderm (ME), neural progenitor cells (NPCs), and mesenchymal stem cells (MSCs). The iHMS [24] database has integrated massive genome-wide epigenetic modification maps and RNA expression data spanning different developmental stages and tissues. From iHMS, we downloaded 6 epigenetic modification maps (H3K4me1, H3K4me3, H3K9me3, H3K27ac, H3K27me3 and H3K36me3) of 20 different human developmental stages and tissues, including hESCs, the hESC-derived precursor cell types (TBL, ME, NPCs and MSCs), and 15 human primary tissues (adipose, adrenal gland, adult liver, aorta, esophagus, gastric, left ventricle, lung, ovary, pancreas, psoas muscle, right ventricle, right atrium, sigmoid colon, spleen, thymus, small intestine, breast, brain and bladder). Meanwhile, the RNA expression data and reference gene annotations were also downloaded from iHMS.

### Overview of the DiffEM model

**Fig. 1:**
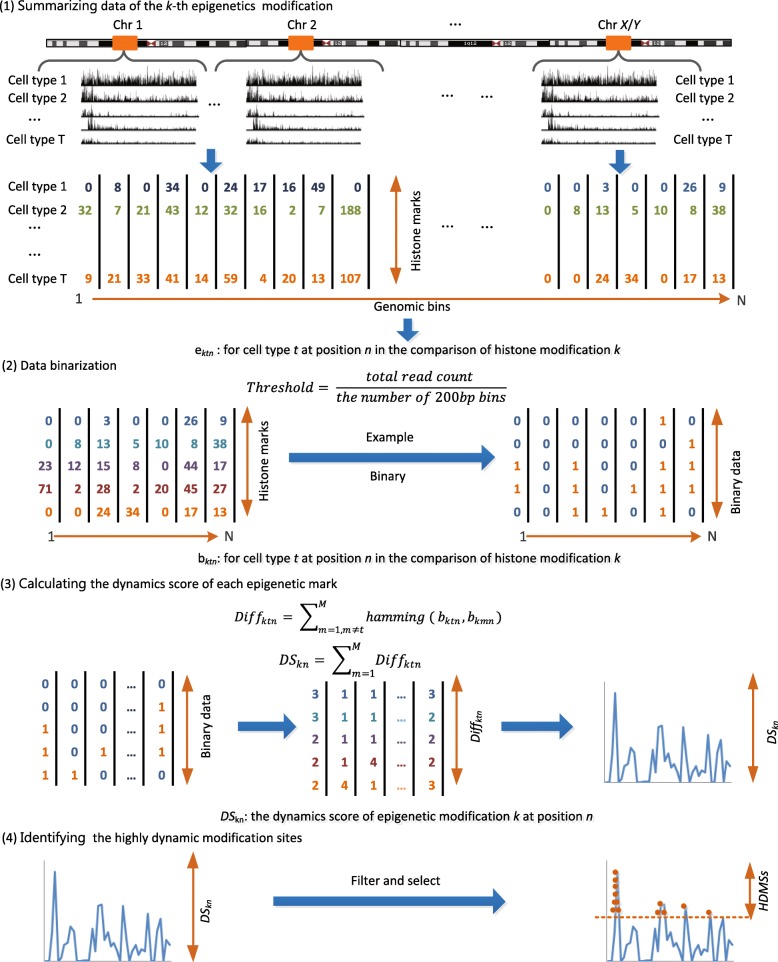
The flowchart of the DiffEM approach

To characterize epigenetic dynamics across different development stages, we developed DiffEM, a new method to estimate the dynamics of epigenetic modifications based on hamming distance and identify highly dynamic modification sites. Unlike the previous work [20], we aimed to detect highly dynamic regions of epigenetic modification during cell differentiation process. To evaluate the dynamics across different differential stages and the primary tissues respectively, these 20 cell types were further categorized into three groups, hESC-derived precursor cell types, primary tissues and the whole group. We introduce the following steps to identify HDMSs, which are also shown in Fig. 1.

*Data binarization.* The raw ChIP-seq data were pre-processed in iHMS database [24]. The whole-genome was first segmented into 200bp bins. For each bin, neighboring read counts were summarized into an integer, indicating the extent of epigenetic modification in this region [25]. To reduce the effect of noise, we transformed these integers into binary values. First, we calculated the binarization threshold, by dividing the total read counts of all bins by the number of bins. If the read count of a bin is higher than the threshold, its binary value is set as 1, otherwise 0, After binarization, we noticed that some bins have no signals in all cell types, which may consist of sequences of low mappability. The consecutive regions with length more than 5 kb were removed from the genome. Finally, for the 6 investigated epigenetic marks, we obtained 6 binary matrices *B*_*K*_ of size *T* (the number of cell types) by *N* (the number of 200 bp bins on the whole genome).

*Calculation of the dynamics scores for each epigenetic mark.* After data binarization, we calculated the dynamics scores for each epigenetic mark among multiple cell types. In particular, we used the hamming distance to measure the dynamics of each epigenetic modification. Here, we respectively calculated the dynamics scores of the 6 investigated epigenetic modifications in three cell type groups. As described above, given *M* cell types and *N* bins, we denoted *b*_*ktn*_ as the binary profiles of epigenetic modification *k* for cell type *t* at position *n*. Then the difference between cell type *t* and others are calculated as:

1 $$ {Diff_{{ktn}}} ={\sum\nolimits}_{m=1,m\neq t}^{M} {hamming (b_{{ktn}}, b_{{kmn}})}  $$

Further, the dynamics score of epigenetic modification *k* at position *n* was summed as:

2 $$ {DS_{{kn}}} ={\sum\nolimits}_{t=1}^{M} {Diff_{{ktn}}}  $$

*Identification of the highly dynamic modification sites.* For each epigenetic mark, we have obtained the dynamics scores along the genome in each cell type group. The higher the dynamics score is, the greater the difference across these cell types exhibits. The sites with zero score were filtered first. Based on the calculated dynamics scores, we selected those bins whose dynamics scores are significantly higher than the genome background (*p* <0.05) and merged the adjacent bins into longer regions. These regions are referred to as highly dynamic modification sites (HDMSs).

### Functional analysis of the highly dynamic modification sites

To investigate the potential functions of these identified HDMSs, we mapped them to RefSeq genes and some functional regions. According to their relative positions, we related the HDMSs to various genes when the centers of HDMSs are located in gene regions. The number of genes related to HDMSs was counted. Furthermore, we mapped the bins with the highest score to genomic features like promoter, coding region and exon. If a HDMS is not related to any gene, it is labeled as an intergenic sites. For further analysis of the functional relevance of HDMSs, we performed gene ontology (GO) enrichment analysis and pathway enrichment analysis for genes enriched with HDMSs via DAVID bioinformatics resources. The significant enrichment lists are obtained with *p*<0.05.

### Comparisons among different epigenetic modifications

Epigenetic modifications play a critical role in cell differentiation process. Different epigenetic modifications may collaborate with each other to execute specific functions. We investigated the relations among different types of epigenetic marks. Based on the identified HDMSs of each epigenetic mark, the common HDMSs between different epigenetic modifications were obtained in the whole genome. Further, we estimated the correlations between the dynamics scores of these epigenetic modifications.

### Correlation analysis between the dynamics of epigenetic mark and gene expression

First, we evaluated the dynamic scores of gene expression along the genome in each cell type group, which was calculated as the variance divided by the mean of gene expression. Then, we evaluated the correlation coefficients between the dynamic scores of epigenetic modifications and gene expression levels. For those identified HDMSs, a higher correlation coefficient indicates that gene expression is more easily regulated by the specific epigenetic modification.

### Comparison among DiffEM, QDMR and IOD

As there exists no gold standard to benchmark highly dynamic modification sites, we adopted an indirect validation strategy. As previous studies [26], the validation was based on the correlations between the dynamics of epigenetic modifications and gene expression levels. To evaluate the performance in identifying HDMSs, we compared DiffEM with existing methods, QDMR and IOD. Unlike the methods restricted to the differential analysis between two cell types, the above three methods are capable of analyzing three or more cell types. QDMR was proposed for genome-wide differential analysis of epigenetic states based on Shannon entropy [22]. IOD was developed to detect differential regions across multiple cell types [27]. We first normalized the epigenetic data, and used QDMR and IOD to detect highly dynamic modification sites. These methods were compared by the correlations between the dynamics of epigenetic modifications and expression levels of the HDMSs.

## Results

To investigate the dynamics of epigenetic modifications during cell differentiation process, we proposed a computational method, DiffEM, to quantify the dynamics score of various epigenetic marks and identify highly dynamic modification sites (HDMSs). We focused on human differentiation-related cell types, consisting of human embryonic stem cell, 4 hESC-derived precursor cell types, and 15 primary tissues. In each cell type, we collected 6 genome-wide epigenetic maps and gene expression datasets. DiffEM was applied to identify HDMSs along cell differentiation process. To evaluate the performance of our proposed method, in this section we analyzed the identified HDMSs to discover their potential biological roles during cell differentiation and development. Furthermore, we compared DiffEM with two previous methods, QDMR and IOD.

### Genome-wide characterization of epigenetic modification dynamics

To better explore the dynamic epigenetic changes across different cell differentiation stages, these 20 cell types were further grouped into three groups, hESCs and hESC-derived precursor cell types, primary tissues and the whole group. For each group and each epigenetic modification mark, we quantified the dynamics score for each bin based on hamming distance, and then ranked these bins according to their dynamics scores. We selected those bins whose dynamics scores were significantly higher than the genome background (*p* <0.05).

**Fig. 2:**
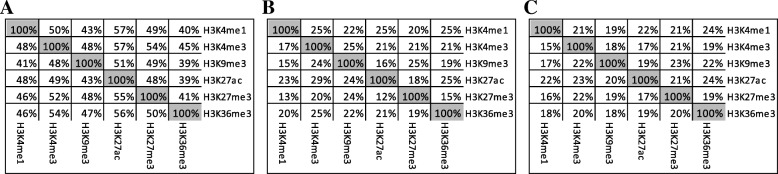
The overlaps between the top HDMSs of different epigenetic marks. The value in row i column j represents the proportion of HDMSs of epigenetic modification i overlapped by those of epigenetic modification j. **a** The hESCs and hESC-derived precursor group. **b** The primary tissues group. **c** The whole group

After merging the neighboring bins, we obtained the HDMSs for each epigenetic modification in each group. For different epigenetic marks, we found that there exist big overlaps between the HDMSs of different epigenetic modifications. This is consistent with previous finding that the epigenetic modifications collaborated with each other to consummate certain regulatory function. As shown in Fig. 2, we respectively calculated the percentage of overlapping HDMSs among 6 epigenetic modifications in these three groups. On the whole, the overlapping sites make up 20%˜60% of total HDMSs in different groups. In the hESCs and hESC-derived precursor group, the HDMSs of different epigenetic marks overlap more than those of the other two groups. For example, the overlap rates of H3K4me1 with other five epigenetic marks range from 40% to 50% in hESC-derived group, while those overlap rates in the other two groups are not greater than 25%. Specifically, H3K4me3 is highly overlapped with H3K9me3 and H3K27ac. These observations demonstrate that epigenetic modifications collaborate closely to regulate the cell differentiation process [4].

**Fig. 3:**
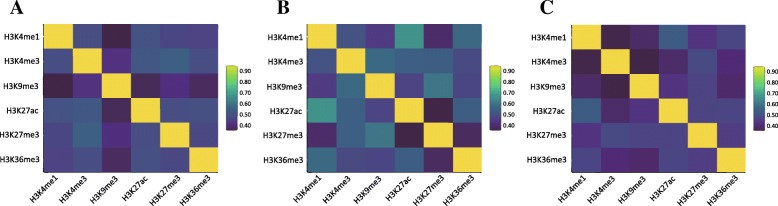
The heatmaps representing the correlations between each pair of epigenetic modifications. **a** The hESCs and hESC-derived precursor group. **b** The primary tissues group. **c** The whole group

As distinct epigenetic modifications share HDMSs, we further investigated the correlation between the dynamics scores of different epigenetic marks. As shown in Fig. 3, the investigated epigenetic marks demonstrate varied correlation in the three comparison groups. In particular, the epigenetic marks show higher correlation in the hESCs and hESC-derived precursor group. This result indicates that the dynamics of epigenetic modifications are similar during the cell differentiation process, which is conformed to the results of previous overlaps analysis.

### Highly dynamic modification sites are related to various genomic features

Further, we mapped the identified HDMSs to RefSeq genes and collected the genes enriched with HDMSs for each epigenetic mark. Here we explored how the dynamic epigenome participates in early embryonic developmental stages and focused on the hESCs and hESC-derived precursor group. To examine the potential functions of those genes, we performed systematic gene ontology enrichment analysis using DAVID tools (https://david.ncifcrf.gov/) and summarized the key biological processes and pathways for each epigenetic mark. Overall, for the aforementioned six epigenetic modification marks, we found that those HDMSs-enriched genes exhibit enrichment for cell differentiation and development functions (Table 1) (*p* value <0.05). For example, GO terms related to development such as ’nervous system development’ are enriched in HDMSs of H3K4me1, H3K4me3, H3K9me3,H3K27ac, H3K27me3, GO terms related to differentiation such as ’neuron differentiation’ and ’cerebellar granule cell differentiation’ are enriched in HDMSs of H3K4me1, H3K9me3, H3K27me3, H3K36me3. This is consistent with previous finding that regulatory elements essential for cellular identity are often epigenetically modified in parental cells [28, 29]. The results highlight the importance of stage-specific epigenetic modification patterns of transcription factors for defining the developmental potentials.

**Table 1:** Functional enrichment of genes on the whole genome of six histone modifications

Term type	Term name	*P*-value	Term type	Term name	*P*-value
H3K4me1					
BP	Cell adhesion	1.42E-06	CC	Cytoskeleton	2.90E-03
BP	Axon guidance	2.86E-05	CC	Growth cone	2.15E-02
BP	Nervous system development	1.91E-04	KEGG	Arrhythmogenic right ventricular	5.82E-03
BP	Signal transduction	1.92E-04		Cardiomyopathy (ARVC)	
BP	Neuron development	2.68E-02	KEGG	Axon guidance	2.79E-02
BP	Cerebellar granule cell differentiation	4.35E-02	KEGG	Hippo signaling pathway	4.32E-02
H3K4me3					
BP	Intracellular signal transduction	7.16E-04	BP	Adult behavior	6.93E-03
BP	Signal transduction	1.22E-03	MF	Extracellular-glutamate-gated ion channel	2.77E-03
BP	Nervous system development	2.63E-03		Activity	
BP	Chemical synaptic transmission	2.88E-02	KEGG	Neuroactive ligand-receptor interaction	6.24E-03
H3K9me3					
BP	Heterophilic cell-cell adhesion	1.87E-07	BP	Regulation of RNA splicing	9.41E-03
BP	Cell adhesion	1.06E-04	BP	Regulation of alternative mRNA splicing	1.58E-02
BP	Nervous system development	6.27E-04	BP	Chemical synaptic transmission	3.35E-02
BP	Regulation of neuron projection	4.09E-03	BP	Cerebellar granule cell differentiation	3.98E-02
	Development		MF	Calcium ion binding	1.25E-05
BP	Signal transduction	5.41E-03	KEGG	Cell adhesion molecules (CAMs)	2.10E-02
H3K27ac					
BP	Signal transduction	7.03E-05	BP	Regulation of RNA splicing	2.08E-02
BP	Nervous system development	7.47E-05	BP	Cytoskeleton organization	3.57E-02
BP	Neuron cell-cell adhesion	2.31E-04	MF	Actin binding	1.20E-04
BP	Neuron development	5.28E-03	CC	Growth cone	1.83E-04
BP	Glutamate receptor signaling pathway	5.76E-03	MF	Protein kinase activity	1.15E-02
BP	Brain development	1.57E-02	KEGG	Neuroactive ligand-receptor interaction	1.06E-02
H3K27me3					
BP	Social behavior	9.15E-05	BP	Cerebellar granule cell differentiation	3.89E-02
BP	Signal transduction	5.10E-04	MF	Calcium ion binding	7.03E-05
BP	Nervous system development	2.97E-03	MF	Cell adhesion molecule binding	1.65E-04
BP	Regulation of RNA splicing	8.99E-03	CC	Growth cone	1.55E-02
H3K36me3					
BP	Heterophilic cell-cell adhesion	4.04E-07	CC	Neuron projection	4.32E-03
BP	Signal transduction	2.27E-03	MF	Actin binding	7.37E-03
BP	Cell adhesion	1.25E-02	KEGG	Neuroactive ligand-receptor interaction	4.97E-02
BP	Neuron differentiation	3.37E-02			

Also, we noticed that the biological processes of distinct epigenetic marks have overlappings. One possible interpretation for this observation could be that these epigenetic marks may have the same changing trend, collaborating with each other to finish the complex regulatory functions. Taken together, the above results of GO annotation demonstrated the power of our method in identifying the highly dynamic sites of these epigenetic modifications. And, the results strongly suggest that the HDMSs mark critical regulatory regions for cell differentiation and development process. Further characterization of epigenetic modification patterns and gene expression within HDMSs may provide important insights into the regulatory functions of the specific epigenetic patterns.

### Highly dynamic modified sites neighboring genes reveal diverse transcriptional patterns

To analyze the regulatory roles of these dynamic epigenetic patterns, we further explored the epigenetic modification and gene expression patterns within HDMSs. We computed the correlation coefficients between the dynamics of epigenetic modifications and gene expression levels of the HDMSs-enriched genes. We mapped the HDMSs to Ref-Seq genes and obtained gene expression of the associated genes. As these 20 cell types were divided into three groups, the dynamics score of gene expression was assessed using the same method as epigenetic marks (see Methods). For those HDMSs located in promoters, and coding regions, the Pearson correlation coefficients were respectively computed.

**Fig. 4:**
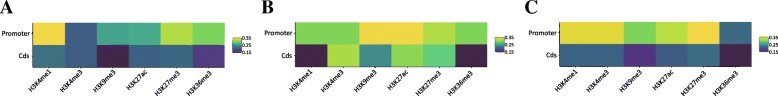
The correlations between the dynamics of epigenetic modification and that of expression level in HDMSs. **a** The hESCs and hESC-derived precursor group. **b** The primary tissues group. **c** The whole group

As shown in Fig. 4, we noted that there is highly correlation between the dynamics of gene expression level and epigenetic modification in promoter regions. Relatively, the correlation in coding regions is lower. These results indicate that the variance of epigenetic modification patterns in promoter regions has a higher regulatory role than that in coding regions. The three different groups have a similar trend. In detail, the six epigenetic modification marks exhibit different regulatory effect. For the hESCs and hESC-derived precursor group, the dynamics of gene expression levels are highly regulated by the modification patterns of H3K4me1 and H3K27me3 in promoter regions. For the primary tissues, the correlations are much higher for H3K9me3 and H3k27ac.

### Comparison with QDMR and IOD in identifying HDMSs

Considering that our method was developed for the differential analysis for multiple cell types, we compared DiffEM with two similar previous methods QDMR and IOD [22, 27], which were also designed for multiple conditions. QDMR is based on Shannon entropy [22], and IOD is defined as the variance divided by the mean value [27]. The performance was measured by the correlation analysis between the epigenetic modification dynamics and gene expression difference.

Firstly, we respectively identified the highly dynamic modification sites using these three methods, and ranked the HDMSs according to the dynamics score. Similarly, we obtained the ranked highly dynamic expression sites. Then, we associated these HDMSs with the highly dynamic expression sites by bitwise matching. To evaluate the performance of these three methods, we define two metrics, *MatchedNum* and *AveDS*. *MatchedNum* is computed as the number of highly dynamic expression sites matching with the top ranked HDMSs, which is similar to recall. *AveDS* represents the average dynamics score of these matched highly dynamic expression sites. Here, for fair comparison among the three methods, we calculated the entropy as the average dynamics score as QMDR.

**Fig. 5:**
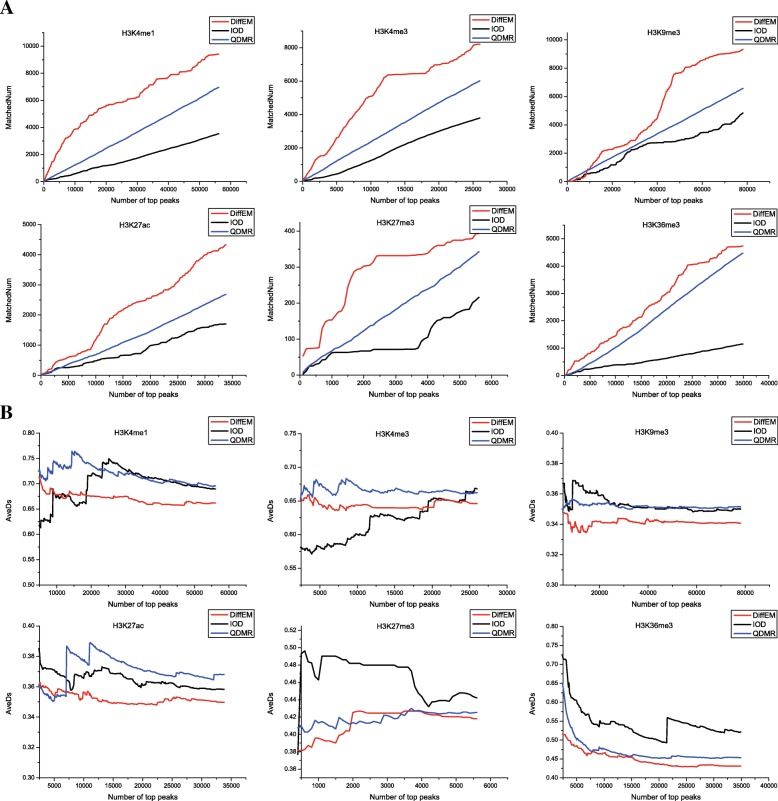
Performance comparisons among our proposed method, IOD and QDMR for each epigenetic mark. The performance was evaluated by *MatchedNum* and *AveDs*. The higher the Num is, and the lower the Ave is, the better the performance in detecting HDMSs. (**a**) The *MatchedNum*, (**b**) The *AveDs*s

We compared the performance on the aforemetioned 6 epigenetic modifications, the results are shown in Fig. 5 and Additional file 1. Figure 5 shows the comparison results for the hESCs and hESC-derived precursor group. Figure S1, Figure S2 (see Additional file 1) showed the results of the other two groups. We first compared the matched numbers of all differential gene expression sites output by these methods. Our method could get a higher *MatchedNum* of highly dynamic expression sites than those of QDMR and IOD (Fig. 5a). However, this raises the question that to what extent these matched sites are dynamically expressed. As we noted that changes in epigenetic modifications could cause differential expression of related genes, we further compared the average dynamics of gene expression of these matched sites. Lower ave indicate better performance. As the results showed (Fig. 5b), our method has good performance in *AveDS*. These observations demonstrate that our method always achieves a balance between matched *MatchedNum* and *AveDS*, which means our approach could be applied to find meaningful HDMSs as many as possible. In addition, the overall analysis for *MatchedNum* and *AveDS* shows that IOD may be applicable to detecting the highest HDMSs, because of the commonly small Num but better *AveDS* of related differential gene expression sites. In summary, our method outperforms the two existing methods in identifying the HDMSs across different developmental stages and tissues in the whole genome.

## Discussion

In this paper, we proposed a new computational method, DiffEM, based on hamming distance to identify the highly dynamic modification sites that undergo chromatin changes during human cell differentiation process. Different from previous methods that mostly focused on differential analysis between two cell types, our method is designed for differential analysis of genome-wide epigenetic modification across multiple cell types. DiffEM can be broadly applied in a range of studies involving various epigenetic marks in different conditions. We applied this approach to investigating 6 epigenetic marks of 20 human cell types, including hESCs, 4 hESC-derived Lineages and 15 human primary tissues. We identified highly dynamic modification sites where different cell types exhibit distinctive epigenetic modification patterns, and found that these highly dynamic modification sites are enriched in the genes are related to cellular development and differentiation. The results also demonstrate the strong association among the dynamics of different epigenetic marks, consistent with previous finding that different epigenetic modifications collaborate with each other to consummate complex regulatory functions. Further, we evaluated the effectiveness of our method, by correlating the dynamics scores of epigenetic modification with the variance of gene expression. We compared DiffEM with two existing methods, QDMR and IOD. The comparison results indicate the power of our method in quantifying the epigenetic dynamics and identifying highly dynamic regions.

## Additional file

Additional file 1 **Figure S1.** Performance comparisons for primary tissues among our method DiffEM, IOD and QDMR for each epigenetic mark. (A) The MatchedNum, (B) The AveDS. **Figure S2.** Performance comparisons for the whole group among our method DiffEM, IOD and QDMR for each epigenetic mark. (A) The MatchedNum, (B) The AveDS. (PDF 405 kb)

## Abbreviations

DAVID: The database for annotation, visualization and integration discovery
GO: Gene ontology
HDMSs: Highly dynamic modification sites
hESCs: Human embryonic stem cells
iHMS: A database integrating human histone modification data across developmental stages and tissues
ME: Mesendoderm
MSCs: Mesenchymal stem cells
NPCs: Neural progenitor cells
TBL: Trophoblast-like cells
