# Supplementary material for: Genome-wide analysis of epigenetic dynamics across human developmental stages and tissues
Source: BMC Genomics. 2019 Apr 4;20(Suppl 2):221. doi: 10.1186/s12864-019-5472-0 (PMC6457072; doi:10.1186/s12864-019-5472-0)
Supplement: Supplementary file 1 — Figure S1. Performance comparisons for primary tissues among our method DiffEM, IOD and QDMR for each epigenetic mark. (A) The MatchedNum, (B) The AveDS. Figure S2. Performance comparisons for the whole group among our method DiffEM, IOD and QDMR for each epigenetic mark. (A) The MatchedNum, (B) The AveDS. (PDF 405 kb) [file 12864_2019_5472_MOESM1_ESM.pdf]

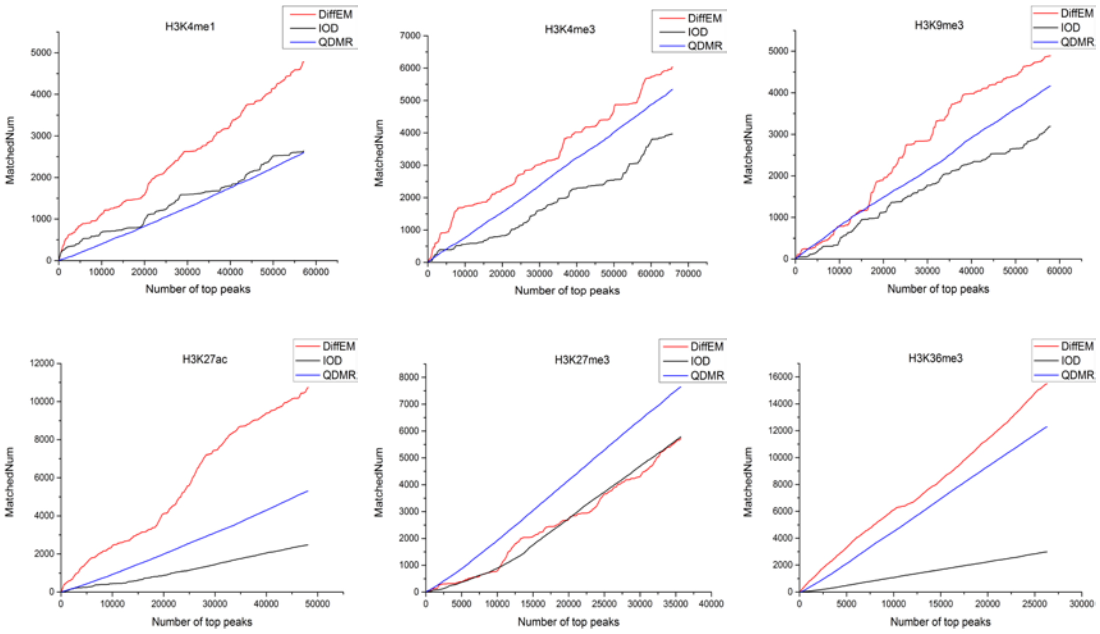

A

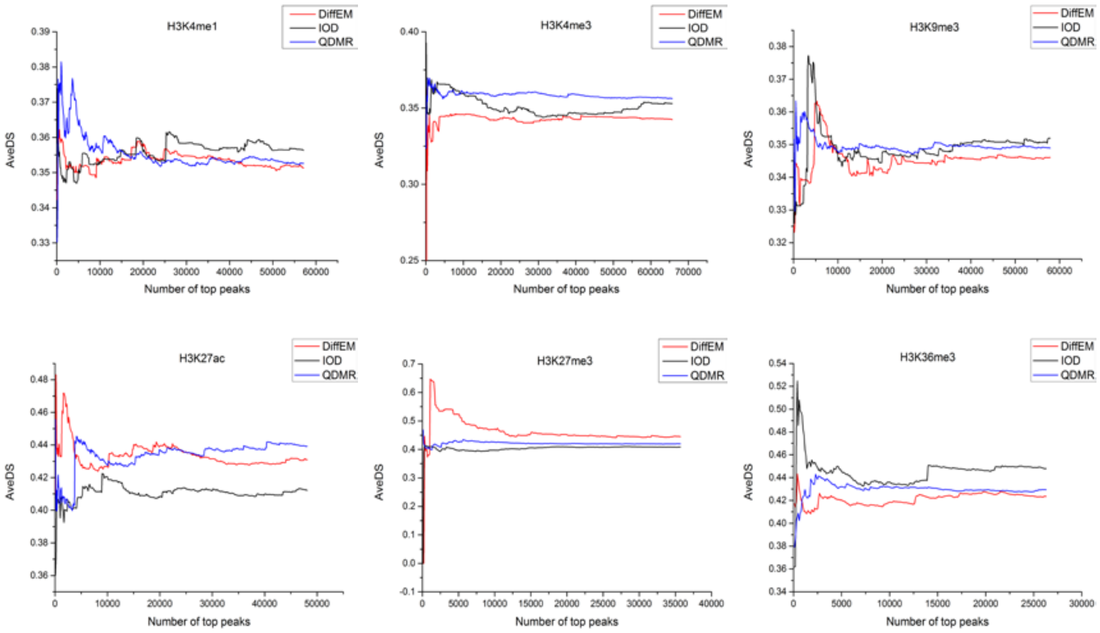

B

Figure S1: Performance comparisons for primary tissues among our method DiffEM, IOD and QDMR for each epigenetic mark. (A) The MatchedNum, (B) The AveDS.

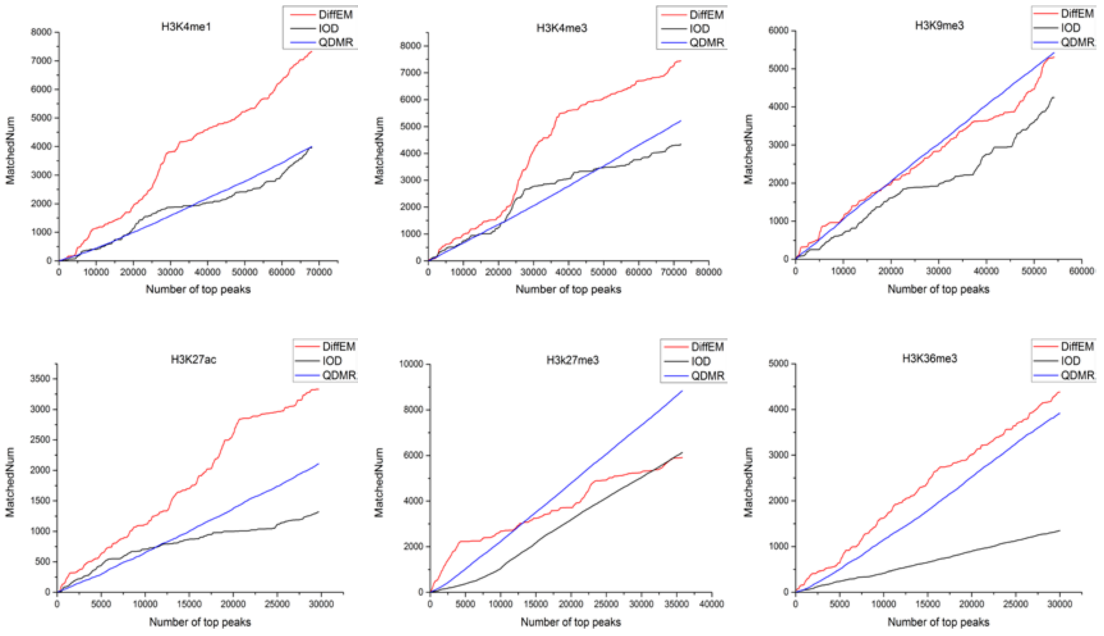

A

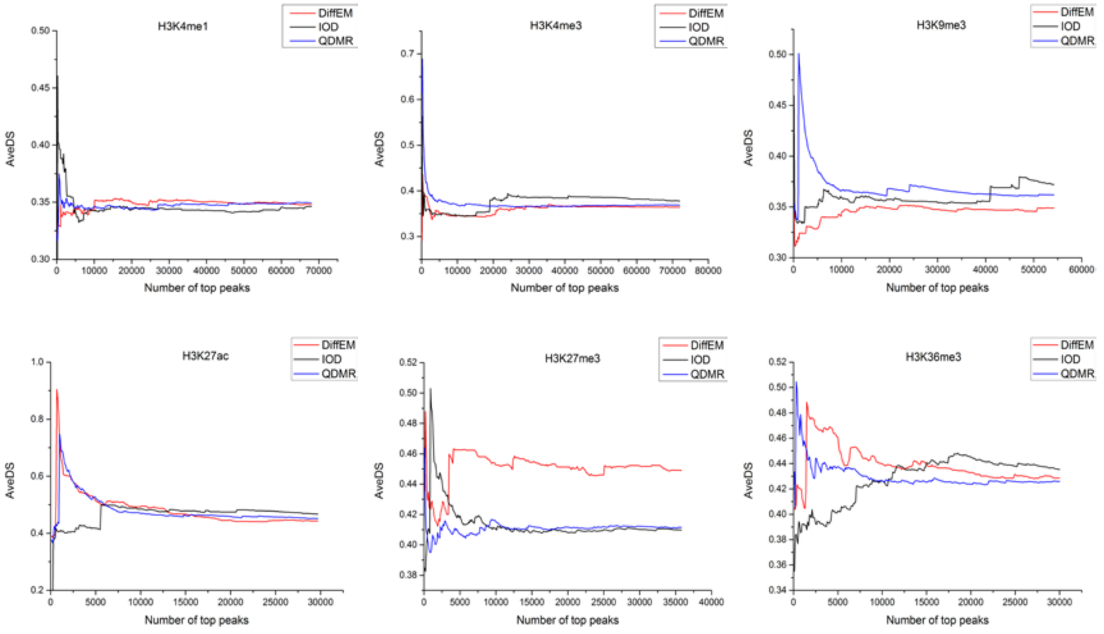

B

Figure S2: Performance comparisons for the whole group among our method DiffEM, IOD and QDMR for each epigenetic mark. (A) The MatchedNum, (B) The AveDS.
